# A pilot study on AI-based voice analysis for monitoring patients hospitalized with acute decompensated heart failure

**DOI:** 10.1093/ehjdh/ztag052

**Published:** 2026-03-30

**Authors:** Leonhard Riehle, Mariam Fouad, Marcus Hott, Emanuel Heil, Chong Bin Lee, Felix Schoenrath, Soumya Vungarala, Bruce Johnson, Lyle Olson, Aurele Goetz, Filipe Barata, Nicholas Cummins, Gerhard Hindricks, Felix Hohendanner

**Affiliations:** Department of Cardiology, Angiology, and Intensive Care Medicine, Deutsches Herzzentrum der Charité, Charitéplatz 1, Berlin 10117, Germany; Noah Labs, Berlin, Germany; Noah Labs, Berlin, Germany; Noah Labs, Berlin, Germany; Department of Cardiology, Angiology, and Intensive Care Medicine, Deutsches Herzzentrum der Charité, Charitéplatz 1, Berlin 10117, Germany; Charité—Universitätsmedizin Berlin, corporate member of Freie Universität Berlin and Humboldt-Universität zu Berlin, Charitéplatz 1, Berlin 10117, Germany; DZHK (German Center for Cardiovascular Research), Partner Site Berlin, Berlin, Germany; Berlin Institute of Health at Charité—Universitätsmedizin Berlin, Charitéplatz 1, Berlin 10117, Germany; Department of Cardiology, Angiology, and Intensive Care Medicine, Deutsches Herzzentrum der Charité, Charitéplatz 1, Berlin 10117, Germany; Charité—Universitätsmedizin Berlin, corporate member of Freie Universität Berlin and Humboldt-Universität zu Berlin, Charitéplatz 1, Berlin 10117, Germany; Department of Cardiothoracic and Vascular Surgery, Deutsches Herzzentrum der Charité, Augustenburger Platz 1, Berlin 13353, Germany; Department of Cardiology, Mayo Clinic, Rochester, MN, USA; Department of Cardiology, Mayo Clinic, Rochester, MN, USA; Department of Cardiology, Mayo Clinic, Rochester, MN, USA; Noah Labs, Berlin, Germany; Centre for Digital Health Interventions, ETH Zurich, Zurich, Switzerland; Department of Biostatistics and Health Informatics, King’s College London, London, UK; Department of Cardiology, Angiology, and Intensive Care Medicine, Deutsches Herzzentrum der Charité, Charitéplatz 1, Berlin 10117, Germany; Charité—Universitätsmedizin Berlin, corporate member of Freie Universität Berlin and Humboldt-Universität zu Berlin, Charitéplatz 1, Berlin 10117, Germany; DZHK (German Center for Cardiovascular Research), Partner Site Berlin, Berlin, Germany; Berlin Institute of Health at Charité—Universitätsmedizin Berlin, Charitéplatz 1, Berlin 10117, Germany; Department of Cardiology, Angiology, and Intensive Care Medicine, Deutsches Herzzentrum der Charité, Charitéplatz 1, Berlin 10117, Germany; Charité—Universitätsmedizin Berlin, corporate member of Freie Universität Berlin and Humboldt-Universität zu Berlin, Charitéplatz 1, Berlin 10117, Germany; DZHK (German Center for Cardiovascular Research), Partner Site Berlin, Berlin, Germany; Berlin Institute of Health at Charité—Universitätsmedizin Berlin, Charitéplatz 1, Berlin 10117, Germany

**Keywords:** Heart failure, Remote monitoring, Vocal biomarker, Voice analysis

## Abstract

**Aims:**

Monitoring pulmonary congestion in chronic heart failure (HF) reduces decompensation and hospitalization, but conventional methods such as weight and symptom tracking are often unreliable. As fluid accumulation affects the lungs and vocal tract, subtle voice alterations may serve as a non-invasive signal for early detection of worsening HF.

**Methods and results:**

The Voice Analysis for Monitoring Patients with HF trial (VAMP-HF, NCT06566911) prospectively enrolled 104 patients hospitalized with acute decompensated HF (ADHF) across two academic centres in the USA and Germany. Daily voice recordings were collected from admission to discharge, with breathing features extracted from speech and acoustic features from sustained vowels. A machine-learning model was trained to classify recordings as admission-phase vs. discharge-phase using leave-one-patient-out. Patients with clinical deterioration, insufficient audio quality, or short length of stay were excluded. Seventy-nine patients were included in the final dataset. The model classified admission and discharge with an *F*_1_-score of 0.83 (95% CI: 0.77–0.90; AUC = 0.90). In patients with higher audio volume (*N* = 54), performance reached 0.89 (95% CI: 0.82–0.94; AUC = 0.91). When applied to intermediate hospitalization days, model-predicted scores showed progressive increases from admission towards discharge. Performance remained robust irrespective of significant weight loss during hospitalization.

**Conclusion:**

In this pilot study, structured voice and breathing analysis discriminated hospitalization phase from admission through discharge in patients with ADHF. This non-invasive approach captured progressive changes during the hospital course and warrants further investigation with concurrent objective congestion markers to establish physiological specificity.

## Graphical Abstract

**Graphical Abstract:**
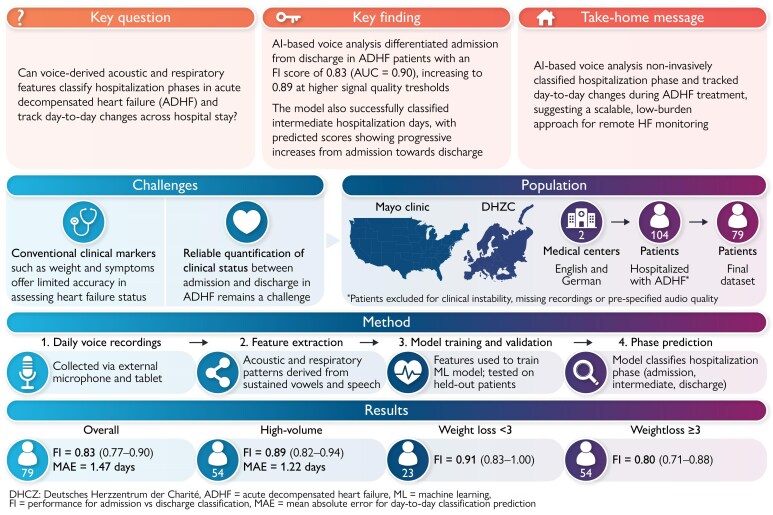

## Introduction

Heart failure (HF) remains a leading public health challenge, affecting approximately 64 million people globally.^1^ Acute decompensated HF (ADHF) accounts for more than one million hospital admissions annually in the United States, with each admission associated with significant clinical deterioration and increased mortality risk.^2^ The trajectory of HF often worsens with repeated hospitalizations, emphasizing the importance of accurate congestion management to prevent subsequent hospitalizations.^3,4^ Remote monitoring has emerged as a potential approach for early detection of worsening HF, allowing timely intervention to mitigate hospitalization risks.^5,6^ Despite wide clinical use, conventional remote monitoring metrics such as daily body weight have demonstrated limited sensitivity in detecting congestion early enough for timely intervention.^7,8^ Prior studies highlight that up to 50% of patients lack significant weight increase prior to HF hospitalization.^9,10^ Volume redistribution from the splanchnic area significantly contributes to ADHF but often remains undetected due to absent absolute weight changes.^11,12^ During inpatient treatment, clinically variable weight measurements and recurring expensive laboratory tests [e.g. *N*-terminal pro-B-type natriuretic peptide (NT-proBNP)] serve as primary discharge indicators.^5^ Novel methods are therefore needed to monitor HF patients in inpatient and outpatient settings.

In response to this unmet need, recent innovations have introduced invasive and non-invasive approaches to assess haemodynamic changes. Implantable sensor-based technologies such as CardioMEMS (Abbott, Inc.) demonstrate effectiveness in reducing hospital readmissions through pulmonary artery pressure-guided remote monitoring.^13,14^ However, invasive approaches remain resource-intensive, costly, and consequently not widely implemented, highlighting the critical need for more accessible monitoring technologies.^5^ Non-invasive remote monitoring programmes based on body weight, symptoms, and vital parameters have produced conflicting results, with large randomized trials failing to demonstrate uniform reduction in cardiovascular outcomes.^15^

Voice and speech analysis has emerged as a promising novel method for non-invasive assessment of physiological changes associated with worsening HF and congestion.^16,17^ Pulmonary congestion, haemodynamic imbalance, and oedema of vocal tract tissues can alter voice production through subtle changes in speech acoustics and breathing patterns.^18,19^ Specific acoustic parameters, including pitch stability, jitter, harmonics-to-noise ratio, and spectral energy distribution, serve as measurable indicators of laryngeal and pulmonary congestion associated with acute HF decompensation.^20,21^ Prior studies demonstrated that voice features change with HF status, but most were confined to small, single-centre cohorts, relied on descriptive speech metrics, and lacked independent validation.^17,19,22^ Murton *et al*. (2023) advanced the field by applying machine learning to daily hospital recordings, yet their analysis remained limited to a single centre and binary ‘early vs. late’ hospitalization classification.^23^ Okada *et al*. showed correlations between acoustic features and concurrent brain natriuretic peptide in hospitalized ADHF patients, yet also in a single centre and language cohort without an independent validation approach.^24^ Consequently, the ability of vocal biomarkers to provide continuous, patient-independent discrimination of hospitalization phases in ADHF remains uncertain.

To address this gap, we designed the Voice Analysis and Monitoring for Predicting HF Decompensation (VAMP-HF) trial. We investigated whether acoustic and respiratory features derived from voice samples can accurately discriminate between admission and discharge states in patients with ADHF when evaluated on held-out patients not used for model training. Additionally, we aimed to determine whether day-to-day changes in clinical trajectory correlate with a voice-based biomarker, and whether performance can be maintained across languages, centres, weight-loss data, and recording devices, thereby enabling widespread outpatient use. We hypothesized that combining voice and breathing features would outperform either modality alone and could provide a scalable, non-invasive digital biomarker for outpatient remote monitoring.

## Methods

### Study design and patient population

The VAMP-HF trial (ClinicalTrials.gov NCT06566911) is a prospective, observational, multicentre cohort study conducted at two tertiary cardiovascular centres: Mayo Clinic (Rochester, Minnesota, USA) and Deutsches Herzzentrum der Charité (DHZC, Germany). Patients hospitalized with ADHF underwent standardized daily voice recordings throughout their inpatient stay, beginning within 24–36 h after admission and continuing until discharge or death.

Eligible patients were adults aged ≥18 years admitted with a primary clinical diagnosis of ADHF confirmed by the attending cardiologist based on clinical signs, symptoms, laboratory markers (elevated natriuretic peptides), imaging (chest X-ray or echocardiography), and clinical assessment in line with current ESC and AHA/ACC/HFSA guidelines.^5,25^ All diagnoses were reviewed by a blinded senior consultant. Patients were eligible regardless of left ventricular ejection fraction (LVEF) phenotype [HF with reduced ejection fraction (HFrEF), mildly reduced ejection fraction (HFmrEF), or preserved ejection fraction (HFpEF)]. Exclusion criteria included: significant cognitive impairment (dementia or Alzheimer’s disease), chronic obstructive pulmonary disease with acute exacerbation or unstable disease, intensive care unit (ICU) or intermediate care (IMC) monitoring requirement, current dialysis therapy, vocal tract surgery history, neurodegenerative conditions (Parkinson’s disease or amyotrophic lateral sclerosis), pregnancy, anticipated non-compliance with daily recordings, or inability to provide informed consent. Patients hospitalized 2 days or fewer, those with clinical deterioration during hospitalization, those with discharge weight exceeding admission weight, and those with insufficient recording quality were excluded from the primary analysis. Clinical deterioration was defined as transfer to an ICU or IMC, need for urgent surgery transfer, or in-hospital death as these events interrupted the planned longitudinal recording protocol and prevented completion of the admission–discharge comparison. Separately, patients whose discharge weight exceeded admission weight were excluded because net weight gain suggests an unclear treatment response that would dilute the admission vs. discharge training signal rather than necessarily indicating clinical deterioration. These exclusion criteria are in line with previous studies^24,26^ and were pre-specified.

The trial received IRB approval from both centres (DHZC: EA2/237/23; Mayo Clinic: 23-011322) and was conducted in accordance with the Declaration of Helsinki and Good Clinical Practice guidelines. All patients provided written informed consent. As this was an exploratory, hypothesis-generating pilot study, the target sample size was based on feasibility of consecutive recruitment and completion of longitudinal voice recordings during hospitalization rather than a formal power calculation.

### Data collection

Clinical data and voice recordings were prospectively collected from admission through discharge. Admission data included NT-proBNP levels (pg/mL), LVEF via echocardiography, baseline demographics, height, and comorbidity history [coronary artery disease, myocardial infarction, coronary artery bypass grafting (CABG), percutaneous coronary intervention (PCI), diabetes, hypertension, chronic kidney disease (CKD)]. Serial NT-proBNP measurements at discharge were not routinely obtained. Daily weight measurements were obtained throughout hospitalization as a simple and readily available marker of longitudinal changes in fluid status. Voice samples were collected each morning at both sites, with additional evening samples at Mayo Clinic. A Sennheiser MKE 600 directional microphone connected to a Zoom H1n recorder, positioned approximately 20 cm from the patient’s mouth, acquired voice data. Recordings were conducted at bedside in ward rooms under standardized conditions. Patients were seated upright or at 45-degree incline (see supplementary material online, *Figure S1*); deviations and environmental factors affecting recording quality were documented. Recording sessions were supervised by clinical investigators.

A standardized protocol of a single recording session contained three speech tasks: sustained vowel phonation, passage reading, and a randomized daily sentence. For sustained vowels, DHZC patients recorded /a/,/i/, and /u/, while Mayo Clinic patients recorded /a/,/i/, and /o/ to align with language-dependent phonetics. Patients were instructed to sustain each vowel for as long as comfortable with most recordings falling within a 5–15 s window. Recordings of any duration were retained for analysis provided they were non-empty. For passage reading, patients read ‘The Grandfather Passage’ (Mayo Clinic) or ‘Nordwind und Sonne’ (DHZC)—established texts providing comprehensive phoneme coverage.^27^ The third task involved reading randomly assigned proverbs (‘Sentence of the Day’) to introduce phonetic variability and prevent habituation bias. The mean recording session duration refers to the total time required to complete the full standardized recording protocol, including sustained vowels, passage reading, and the sentence-of-the-day. All recording sessions were segmented by task and underwent manual quality assessment and annotation.

### Features extraction

Audio signals were recorded in stereo at 96 kHz sampling rate and 32-bit depth using signed 24-bit PCM encoding. Only sustained vowels and breathing segments extracted from sentence-level tasks (poems and random sentences) were retained for analysis. A customized breathing segmentation pipeline extracted breathing segments [Supplementary material online, Appendix A1, breathing extracted features include inspiratory rise time (time for amplitude envelope to increase from 10% to 90% of peak; Supplementary material online, Appendix A3)]. Pre-processing comprised: (1) low-pass filtering (24 kHz cut-off) to attenuate ultrasonic noise while preserving perceptually salient spectral details relevant to vowel timbre^28,29^; (2) trimming of leading/trailing silences; and (3) amplitude normalization to [−1, 1] to reduce inter-sample variability.

Acoustic features were extracted using OpenSMILE toolkit (eGeMAPsv02 and emobase). Feature sets included low-level descriptors [MFCCs, *F*_0_, intensity, jitter, shimmer, loudness, spectral/cepstral measures (e.g. spectral flux (measure of how rapidly frequency components change), voice quality parameters] and their first-order derivatives, summarized using statistical functionals (mean, SD, min, max) to produce fixed-length representations. This feature set has been validated in clinical and paralinguistic research, including Alzheimer’s disease detection and broader paralinguistic studies.^27–32^

### Dataset construction and sensitivity analyses

Vowel recordings were segmented into non-overlapping 100 ms frames, consistent with their quasi-stationary properties. Breathing recordings were analysed in their entirety to preserve temporal and spectral structure. Both were windowed with a Hanning function to reduce spectral leakage. Segments with invalid *F*_0_ values (*F*_0_ = 0) were excluded. Only tasks available at both admission and discharge were retained per patient. Metadata (age, sex, audio task) were incorporated as auxiliary features. Features exhibiting variance <0.01 and Pearson’s |*r*| > 0.95 were removed to reduce redundancy. Subgroup analyses assessed effects of weight loss, language, and task type by applying the trained model directly to respective data partitions without retraining. Additionally, patients were stratified by recording volume (threshold > −35 dBFS) to assess the impact of technical signal quality on model performance; as all recordings undergo per-recording amplitude normalization, this stratification reflects recording quality rather than a clinical variable (see Supplementary material online, Appendix A2).

To evaluate cross-device generalizability, 10 Mayo Clinic patients completed parallel recordings using simultaneously the professional system and a consumer tablet (Samsung Galaxy Tab S9 FE+) running the Noah Labs application. Features were extracted identically from both devices. To assess device-related differences, matched recordings were aggregated by calculating feature-wise means, and statistical comparisons used Wilcoxon signed-rank tests with false discovery rate (FDR) correction (see Supplementary material online, Appendix A4). For model-level evaluation, the model was trained exclusively on professional-device recordings from patients without dual setups and tested on tablet recordings from dual-setup patients entirely withheld from training (cross-device direct testing).

Finally, to assess cross-site generalizability, we performed a site-held-out evaluation in which all recordings from one clinical site were used exclusively for model training and validation, while all recordings from the second site were reserved as a fully blinded external test set. Consequently, two complementary experiments were conducted: training on Mayo clinic and testing on DHZC, and vice versa. No patients, recordings, or metadata from the held-out site were used at any stage of model training, feature selection, or hyperparameter tuning.

### Statistical analysis and machine learning analysis

Paired samples were compared using Wilcoxon signed-rank tests (two-sided). Effect size was expressed as Cohen’s *d* for dependent samples^33^:


$$\mathrm{cohen}\_d=\frac{\left({\bar{X}}_{\mathrm{admission}}-{\bar{X}}_{\mathrm{discharge}}\right)}{s_{d}},\;\mathrm{where}\;s_{d}=\sqrt{\left(\frac{1}{N-1}\right)\times \Sigma_{i=1}^{N}{\left(d_{i}-\bar{d}\right)}^{2}}$$

where ($d_{i}$) is the within-subject difference and ($s_{d}$) its standard deviation. Conventional thresholds of 0.2, 0.5, and 0.8 were interpreted as small, medium, and large effects, respectively.^33^ Benjamini–Hochberg FDR controlled for multiple comparisons; adjusted *P* < 0.05 was considered significant.^34^

Patient data were represented as temporal sequences with admission and discharge days assigned ordinal relevance scores; intermediate days were linearly ordered by time since admission. The model was trained using nDCG (normalized Discounted Cumulative Gain) ranking objective. To avoid information leakage during training and testing, all model evaluations were performed in a leave-one-patient-out (LOPO) framework such that all recordings from the held-out patient were excluded from training. This approach resulted in a series of models, each evaluated on a patient whose data were not included in the corresponding training set, thereby ensuring independence between training and test data (‘unseen patient’). No external datasets or pretraining were used, and model hyperparameters were fixed across all folds. The machine-learning model generated predictions at the recording segments level, however, test performance was reported at the patient level by aggregating multiple recordings per patient per timepoint using the *F*_1_-score and the percentage of correctly classified samples. Patient-level correctness was defined *a priori* to account for multiple recordings per admission and discharge. Recording-level model scores were aggregated per time point using two complementary measures of central tendency (mean and median) to accommodate potential skew and outliers. A patient was considered correctly ranked if discharge exceeded admission under either aggregation (OR/max-rule decision fusion).^35^ This definition was pre-specified and applied uniformly across all patients. The model was trained on a multilingual dataset without language stratification.

Continuous variables are reported as mean ± SD or median (IQR). Categorical data are presented as counts and percentages. 95% CIs for proportions were calculated using patient-level (cluster) bootstrap resampling (1000 samples) to account for within-patient correlation from repeated recordings; the 2.5th and 97.5th percentiles defined the CI. Analyses were performed using Python 3.10 with SciPy 1.6.1 and XGBoost 2.1.3. This study is reported in accordance with the Strengthening the Reporting of Observational Studies in Epidemiology (STROBE) statement; a completed checklist is provided in Supplementary Material A6.

## Results

Between 1 August 2023 and 10 June 2025, 104 ADHF patients were enrolled. Nine were excluded for recording quality issues (background noise,^8^ extremely low volume,^1^) eight for insufficient hospital duration (≤2 days), five for clinical deterioration, and three because discharge weight exceeded admission weight. The final analysis set comprised 79 patients, 46 from Charité (German-speaking), and 33 from Mayo Clinic (English-speaking). Mean recording session duration was 54.4 s.

The population was predominantly male (70.9%), elderly (mean age: 72.5 ± 14.1 years), with high comorbidity burden: hypertension (76%), CKD (60.8%), diabetes, and prior ischaemic heart disease were common. Admission NT-proBNP was markedly elevated (9433.4 ± 10478.2 pg/mL). Mean hospital stay was 6.62 days (±3.4) reflecting the heterogeneous clinical course and the median time from hospital admission to the first voice recording was 26.9 h. Detailed baseline characteristics of included and excluded patients appear in *Table 1*. Demographic and clinical baseline variables (age, sex, LVEF, admission NT-proBNP, and comorbidity prevalence) did not differ significantly between groups. As expected, excluded patients had shorter hospital stays (3.84 ± 1.80 vs. 6.62 ± 3.41 days, *P* < 0.001) and less weight loss (2.59 ± 2.77 vs. 4.96 ± 4.53 kg, *P* = 0.006), consistent with the applied exclusion criteria.

**Table 1:** Baseline characteristics of the derivation and excluded cohorts

	Derivation cohort (*N* = 79)	Excluded cohort (*N* = 25)	*P*-value
Sex, male	56 (70.9%)	18 (72%)	1.0
English-speaking, *n* (%)	33 (41.8%)	16 (64%)	0.09
Age, years	72.47 ± 14.17	73.52 ± 15.30	0.82
Body-weight change	4.96 ± 4.53	2.59 ± 2.77	0.006
Weight reduction ≥3 kg, *n* (%)	55 (69.6%)	9 (36%)	0.006
Weight reduction in patients ≥3 kg, kg	6.98 ± 3.48	5.42 ± 1.98	0.30
Length of stay on peripheral ward, days	6.62 ± 3.41	3.84 ± 1.80	<0.001
LVEF, %	40.79 ± 16.84	42.43 ± 14.72	0.74
HFrEF (EF ≤ 40%), *n* (%)	37 (46.8%)	10 (40%)	0.96
HFmrEF or HFpEF (EF > 40%), *n* (%)	42 (53.1%)	13 (52%)	
NT-proBNP at admission, pg/mL	9433.43 ± 10478.19	7366.08 ± 8554.29	0.27
Serum creatinine at admission, mg/dL	1.56 ± 0.64	1.81 ± 0.87	0.35
Alanine aminotransferase at admission, U/L	55.11 ± 106.60	63.94 ± 121.70	0.88
Aspartate aminotransferase at admission, U/L	79.63 ± 240.93	88.66 ± 176.19	0.71
Height, cm	173.21 ± 9.59	172.23 ± 11.14	0.82
Patients with chronic heart disease, *n* (%)	45 (57.0%)	18 (72%)	0.18
History of myocardial infarction, *n* (%)	15 (19.0%)	10 (40%)	0.34
History of diabetes, *n* (%)	27 (34.2%)	11 (44%)	0.83
History of CABG/PCI, *n* (%)	25 (31.7%)	9 (36%)	0.31
History of hypertension, *n* (%)	60 (76.0%)	23 (92%)	0.82
History of CKD, *n* (%)	48 (60.8%)	18 (78%)	1.0

CABG = coronary artery bypass grafting; CKD = chronic kidney disease; HFrEF = HF with reduced ejection fraction; HFmrEF = HF with mildly reduced ejection fraction; HFpEF = HF with preserved ejection fraction; LVEF = left ventricular ejection fraction; NT-proBNP = *N*-terminal pro-B-type natriuretic peptide; PCI = percutaneous coronary intervention. Baseline characteristics of included (*N* = 79) and excluded (*N* = 25) patients. Continuous variables are shown as mean ± SD, categorical variables are *n* (%). *P*-values from Wilcoxon rank-sum tests (continuous) and chi-square tests (categorical). Body-weight reduction refers to net weight loss during hospitalization. Left-ventricular ejection fraction categories follow the 2021 ESC guidelines (5). LVEF data unavailable for two excluded patients.

**Figure 1:**
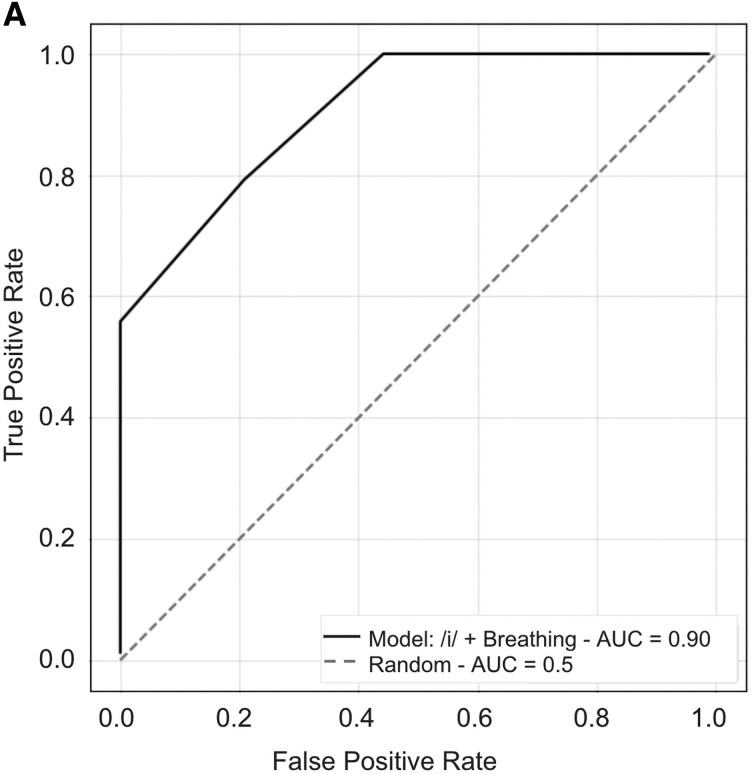
Discrimination of admission and discharge states using vowel- and breathing-based voice analysis. (*A*) Receiver operating characteristic (ROC) curves for the best-performing model (vowel /i/ + Breathing segments) on all patients (*N* = 77) depicting the ability of the model to distinguish between admission and discharge samples.

**Figure 2:**
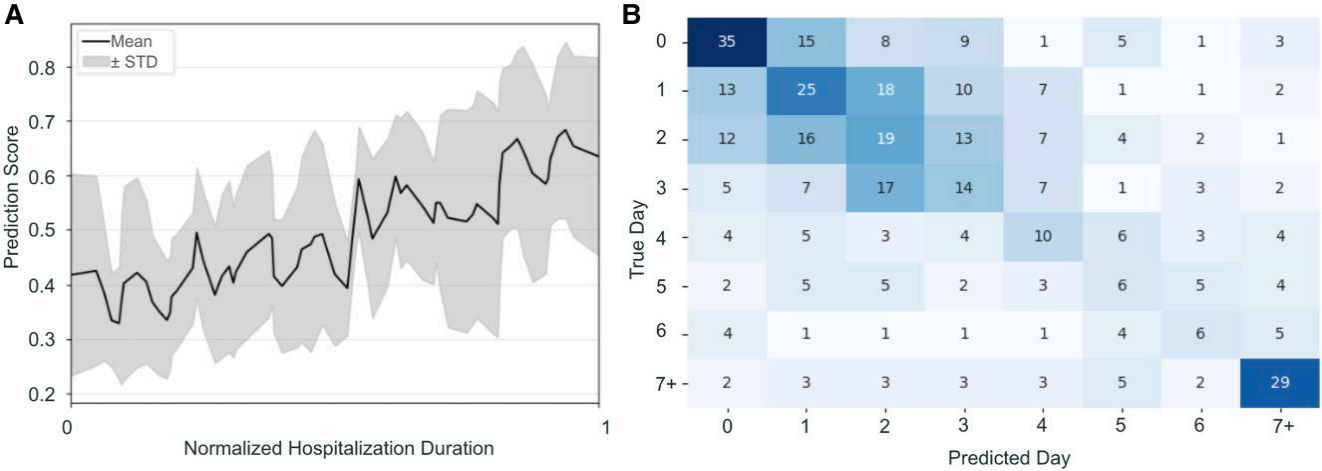
Generalization performance of the speech-based model to unseen intermediate hospitalization days using a leave-one-patient-out evaluation on all patients. (*A*) Mean transformed patient-level prediction scores (sigmoid *z*-scores) across patient-specific normalized hospitalization duration (0 = admission, 1 = discharge), smoothed with a Gaussian filter. The prediction scores continuously increase throughout the hospitalization stay. Shaded area represents ±1 standard deviation across patients. (*B*) Confusion matrix comparing true vs. predicted hospitalization days for held-out patients. The model was trained exclusively on recordings from admission and discharge days and then evaluated on intermediate days for the left-out patient (*N* = 77).

As demonstrated in *Figure 1*, combining sustained vowel /i/with breathing segments, the model yielded a patient-level *F*_1_-score of 0.83 (95% CI: 0.77–0.90) and AUC 0.90 in distinguishing admission from discharge. In patients with higher recording volume (*N* = 54, threshold > −35 dBFS,^36^  Supplementary material online, Appendix A2), performance reached 0.89 (95% CI: 0.82–0.94). The model successfully classified intermediate hospitalization days, with prediction scores steadily increasing from admission to discharge (*Figure 2*). Mean absolute error of daily predictions was 1.47 days overall and 1.22 days for high-volume recordings (*N* = 54). Hospital day was correctly predicted within ±1 day for 63.6% (all) and 66.6% (high-volume) of recordings and within ±2 days for 78.3% and 82.0%, respectively.

**Figure 3:**
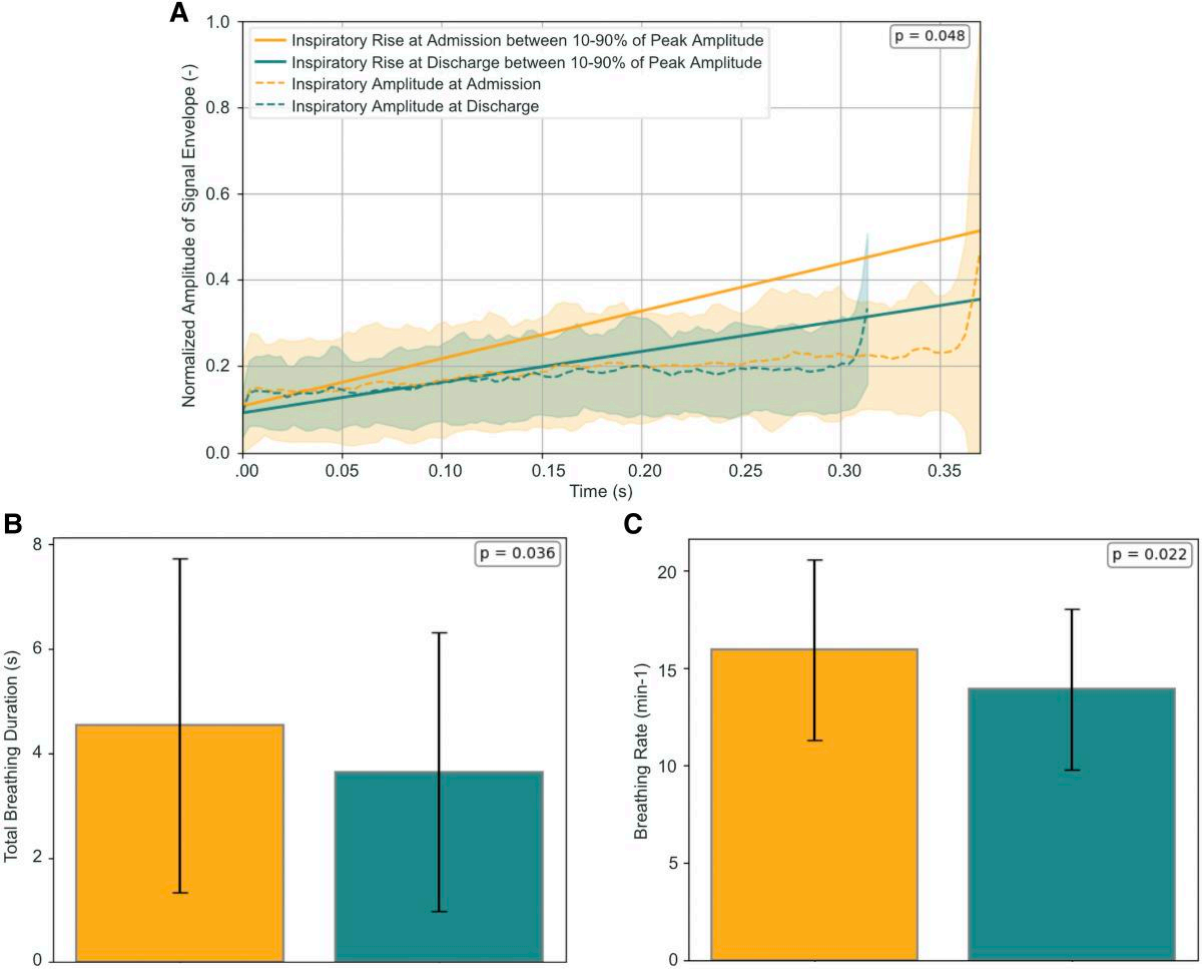
Respiratory acoustic features comparing hospital admission and discharge recordings. (*A*) Mean inspiratory airflow dynamics at admission (orange) and discharge (teal). Solid lines depict the mean linear slope of inspiratory airflow rise, defined as the interval during inspiration where the amplitude envelope increases from 10% to 90% of its peak. Dashed lines represent the mean envelope amplitude across all patients at each timepoint, normalized to the individual recording’s amplitude range. Shaded regions illustrate ±1 standard deviation (see Supplementary material online, Appendix A3). (*B*) Mean total breathing duration (seconds) derived from standardized poem recordings at admission and discharge. (*C*) Mean breathing rate (number of breaths per minute) derived from the same recordings at admission and discharge.

Analysis of 1373 inspiratory segments revealed consistent acoustic changes across hospitalization phases. Rise time for inspiratory airflow was longer at admission and became significantly shorter at discharge (*Figure 3A*). Inspiratory rise slope was steeper at admission (*P* = 0.04), suggesting a more abrupt airflow onset in decompensated state at admission. Total breathing duration (*Figure 3B*) and breathing rate (*Figure 3C*) decreased from admission to discharge (4.5 vs. 3.7 s and 16 vs. 14 breaths per minute, respectively).

Sustained vowel analysis showed marked improvements over hospitalization. Frequency variation (jitter, *P* < 0.01, Cohen’s *d* = 0.3) and amplitude variation (shimmer, *P* = 0.049, Cohen’s *d* = 0.2) declined from admission to discharge. Loudness SD as a measure of vocal intensity variability and spectral flux also decreased towards discharge (*P* < 0.01 for both; Cohen’s *d* = 0.4 and 0.3, respectively). Effect sizes were generally small to moderate.

**Figure 4:**
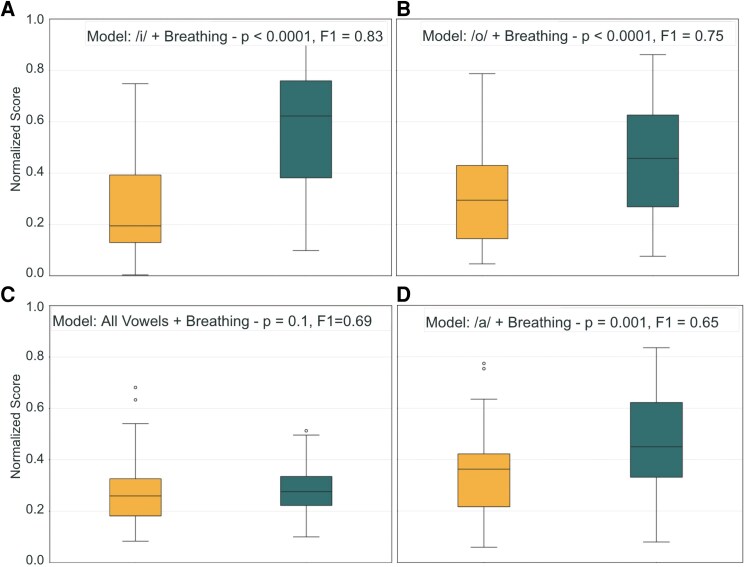
Sensitivity analysis—effect of used vowel: (*A–D*) box plots show the normalized model-predicted scores at admission (orange) and discharge (teal) across all patients (all vowels: *N* = 79, vowel/i/: *N* = 77, vowel/o/: *N* = 75 and vowel/a/: *N* = 79). Per patient scores are computed as an average of all daily recordings. Statistical significance between admission and discharge distributions as well as *F*_1_-score for the models is indicated.

As shown in *Figure 4*, not all vocal tasks contributed equally. Vowel /i/combined with breathing yielded the highest accuracy (*F*_1_ = 0.83, 95% CI: 0.77–0.90, *P* < 0.0001). Vowels /o/ and /a/ performed slightly worse (*F*_1_ = 0.75 [95% CI: 0.67–0.82, *P* < 0.0001] and 0.65 [95% CI: 0.56–0.73, *P* = 0.001], respectively); aggregating vowels yielded *F*_1_ = 0.69 (95% CI: 0.62–0.77, *P* = 0.1); breathing alone yielded *F*_1_ = 0.62 (95% CI: 0.53–0.71, *P* = 0.04).

The model correctly classified hospitalization phase in 83% of patients overall, independent of weight loss magnitude. Patients with weight loss of <3 kg had *F*_1_ = 0.91 (95% CI: 0.83–1.00, *N* = 23); those with ≥3 kg had *F*_1_ = 0.80 (95% CI: 0.71–0.88, *N* = 54). Language performance: German *F*_1_ = 0.74 (95% CI: 0.64–0.85, *N* = 46); English *F*_1_ = 0.97 (95% CI: 0.94–1.00, *N* = 31). In a post-hoc exploratory analysis, the model was applied to the patients excluded from the training cohort, with adverse or unclear clinical trajectories (*N* = 7). The progressive admission-to-discharge pattern observed in the primary cohort was not identified in these patients (see Supplementary material online, *Table II*, Supplementary material online, Appendix A5).

Cross-device comparison (*N* = 10, matched tasks) revealed no significant differences for key predictive parameters: *F*_0_ (*P* = 0.78), spectral flux (*P* = 0.72), shimmer (*P* = 0.16), jitter (*P* = 0.088) (see Supplementary material online, Appendix A4, Supplementary material online, *Table I*). In cross-device direct testing, the model achieved *F*_1_ = 0.68 (95% CI: 0.55–0.98) on unseen patients and devices; restricted to a higher-volume cohort (*N* = 54), *F*_1_ = 0.77 (95% CI: 0.58–0.99).

In cross-site out-of-distribution evaluation, the model demonstrated cross-domain generalization at the patient level. Training on Mayo Clinic and testing on DHZC yielded an *F*_1_ score of 0.67 (95% CI: 0.56–0.76), while training on DHZC and testing on Mayo Clinic resulted in an *F*_1_ score of 0.85 (95% CI: 0.76–0.95).

## Discussion

To our knowledge, this study is the first to demonstrate that short daily voice recordings can discriminate admission, intermediate, and discharge hospitalization phases in ADHF patients, with evaluation on entirely unseen patients. Using breathing-derived features combined with sustained vowels, our model achieved *F*_1_ = 0.89 in a high-volume subset for admission vs. discharge classification. Vowel /i/ yielded highest accuracy, with other vowels or breathing segments alone yielding lower performance. Notably, the model also successfully classified intermediate hospitalization days with model-predicted scores showing progressive increases from admission towards discharge.

A central consideration for interpreting these results is that our model classifies recordings based on their proximity to admission vs. discharge, rather than against validated objective markers of cardiac congestion. This approach is consistent with prior voice biomarker studies in ADHF, including the work by Amir *et al*., which similarly used admission and discharge as reference timepoints.^19^ Importantly, admission necessity and discharge readiness in our study were determined by treating physicians not involved in this investigation in accordance with current ESC and AHA/ACC/HFSA guidelines.^5,25^ While these clinical endpoints reflect standard-of-care decision-making and are the basis for real-world patient management, they do not provide direct physiological validation of congestion status. The acoustic changes observed may therefore reflect multiple factors that differ between admission and discharge, including fluid status, respiratory effort, fatigue, or other aspects of clinical recovery. While we interpret these findings as consistent with clinical improvement during decongestion therapy, alternative explanations cannot be excluded.

Quantifying HF status remains a key clinical challenge.^37^ Residual congestion is a major risk factor for early readmission, with rates reaching 30% within 90 days post-discharge and showing increases in recent years.^38–40^ Prolonged inpatient stays negatively affect outcomes and increase healthcare costs. Conventional bedside assessment has well-documented sensitivity and specificity limitations.^41,42^ Our findings demonstrate that voice analysis offers a potentially objective, non-invasive approach to discriminate hospitalization phase and to track clinical trajectory during inpatient treatment.

Prior single-centre studies demonstrated that voice features change with HF status and can separate admission from discharge, but without independent validation.^19,22^ Murton *et al*. (2023) applied machine learning but remained limited to single-centre and binary classification.^23^ Okada *et al*. demonstrated correlations between acoustic features and concurrent BNP levels during ADHF hospitalization, while Sara *et al*. showed significant voice differences in a large outpatient chronic HF cohort, supporting potential applicability in ambulatory settings.^24,43^ Our findings confirm and extend these observations. We demonstrated strong admission–discharge separability on patient-independent folds and showed that model-predicted scores correlate with hospitalization day (mean absolute error of 1.47 days), though this reflects temporal correlation rather than direct measurement of physiological recovery. Combining respiratory acoustics with vowel features boosted performance.

The pathophysiology of voice changes in HF is plausible yet not fully established. Pulmonary congestion leads to fluid accumulation in interstitial and alveolar compartments, impairing airflow and vocal-fold mechanics.^44,45^ Elevated alveolar fluid and interstitial oedema may increase airway resistance and turbulence, potentially disrupting laminar inspiratory airflow. Laryngeal and vocal-fold micro-oedema are presumed to alter vibratory characteristics.^44^ Our results support this framework, showing acoustic instability reductions and increased inspiratory rise times at admission that decrease significantly during the hospital course. Combining breathing parameters with voice-derived features captured multidimensional aspects of recovery, enhancing performance while providing mechanistic interpretability linking acoustic changes to respiratory effort, airflow stability, and dyspnoea.

Importantly, the present study cannot determine the specific physiological mechanism underlying the observed admission–discharge acoustic differences. Although congestion relief is a plausible contributor, alternative correlates of hospitalization phase may also influence the signal. These might include treatment effects not directly related to decongestion (e.g. rate or rhythm control, afterload reduction), exertion during the acute admission period, improved patient cooperation at discharge, supplemental oxygen use, or other pharmacological interventions. Accordingly, the present findings represent hospitalization-phase discrimination; establishing a direct link to specific physiological mechanisms will require further study with concurrent objective congestion measures. However, when the trained model was applied to excluded patients who did not follow a typical improvement trajectory, the expected progressive admission-to-discharge pattern was absent, consistent with their adverse or unclear clinical courses (see Supplementary material online, *Table II*). While these subgroups are small, the findings motivate future investigation into whether atypical or adverse clinical courses can be detected through voice analysis, which would be a prerequisite for clinical actionability in real-world monitoring.

Notably, our model demonstrated robust performance in patients without significant weight loss, supporting that selected features might represent a generalizable attribute of ADHF hospitalization trajectories irrespective of weight change during the hospitalization period. Patients losing <3 kg showed the same direction and magnitude of acoustic improvement as those with more pronounced weight loss. To our knowledge, no prior voice biomarker studies have stratified performance by weight change, making this the first demonstration that acoustic recovery signals can be detected independently of conventional weight-based metrics. These findings align with haemodynamic studies showing that modest diuresis can relieve filling pressures and reduce extravascular lung water before large weight changes occur.^46^ Redistribution of fluid from central circulation to periphery, decreases in pulmonary capillary pressure, and resolution of laryngeal mucosal micro-oedema can improve airflow mechanics and voice production without producing large net weight changes.^11,23^ Voice- and breathing-derived features therefore capture these early, compartment-specific improvements not reflected on the scale, reinforcing their clinical value even in patients with minimal weight loss.

Our sensitivity analysis revealed vowel /i/ consistently outperformed/o/and/a/across languages. We speculate this relates to its articulatory characteristics—high tongue placement and vocal-tract narrowing—that emphasize airflow restriction and vocal-fold vibration.^43^ Although previous studies suggested /i/ may yield superior performance, this work first demonstrates this effect in a HF-specific voice-analysis model.^44^ Aggregating vowels did not improve accuracy, suggesting indiscriminate combination introduces variability reducing discriminative power. Breathing segments alone were relatively weak predictors, yet substantially boosted performance when paired with /i/, indicating complementary information from speech and respiration. These findings suggest targeted selection of highly informative tasks provides greater diagnostic value than aggregating across all tasks, while offering favourable balance between analytical sensitivity and patient feasibility.

Previous voice-based HF studies relied on cross-sectional analysis or binary comparisons lacking temporal resolution.^19^ Our approach advances the field by capturing daily trajectory of clinical improvement and demonstrating that voice-based models distinguish not only extreme states but also intermediate, clinically relevant recovery changes. These results were obtained on a fully independent test set comprising unseen patients, recordings, and states, ensuring generalizability. The enhanced temporal resolution has major clinical implications for supporting treatment adjustment during hospitalization and potentially extending to outpatient remote monitoring. This continuous, low-burden, non-invasive approach could identify deterioration or improvement with high sensitivity—a frequent limitation of conventional HF monitoring.^13,47^ Major remote monitoring trials (TIM-HF, Tele-HF, BEAT-HF) failed to improve outcomes using weight and symptoms with sensitivity as low as 10–20%.^48^ Although pulmonary artery pressure-guided monitoring has been shown to reduce HF hospitalizations, its invasive and costly nature limits widespread use across broader HF populations.^13,14^ Abraham *et al*. postulate that AI-based voice analysis can address these limitations through non-invasive, scalable cardiopulmonary assessment.^47^

Our parallel recording sub-study supports this potential, demonstrating the robustness of key predictive parameters across recording setups. The absence of significant feature differences between high-fidelity recorders and standard consumer tablets indicates voice monitoring might be feasible using readily available devices, supporting practical feasibility and scalability. Cross-device direct testing achieved *F*_1_ = 0.68 (95% CI: 0.55–0.98), with improved performance in higher-volume recordings (*F*_1_ = 0.77, 95% CI: 0.58–0.99). However, these results should be interpreted as preliminary feasibility evidence only. The wide confidence intervals reflect the small sample size (*N* = 10), and the reduction in performance compared with professional-device recordings indicates that cross-device robustness is not yet established. These findings do not support conclusions regarding deployment readiness; future work must evaluate robustness across heterogeneous consumer devices, microphone characteristics, and realistic ambient noise in outpatient conditions.

To address whether acoustic biomarkers generalize across institutions, languages, and clinical practices, we performed cross-site out-of-distribution evaluation. To our knowledge, this is the first such analysis in voice-based HF monitoring. In this dataset, language and clinical site are fully collinear (all DHZC recordings are German; all Mayo Clinic recordings are English), and the cross-site evaluation therefore reflects a combined domain shift encompassing language, recording environment, and clinical workflow. Despite this, the model maintained discriminative performance when trained entirely on one centre and tested on the other, suggesting that the captured acoustic features are not purely site-specific artefacts. The asymmetry in performance between directions (*F*_1_ = 0.67 when training on Mayo and testing on DHZC vs. *F*_1_ = 0.85 in the reverse) may reflect differences in treatment intensity, length of hospitalization, phonetic structure, ambient ward acoustics, case mix, and the larger DHZC training set (*n* = 46 vs. *n* = 33).

To establish physiological specificity and clinical utility, key next steps include prospective studies with concurrent objective congestion markers (serial natriuretic peptides, lung ultrasound B-line quantification, and invasive haemodynamic assessment) and pre-specified clinical endpoints such as worsening HF events and hospitalizations. These studies should be conducted in prospective outpatient cohorts under realistic conditions, including device heterogeneity and unsupervised self-recording.

## Study limitations

While multicentre and multilingual, our modest sample size and controlled inpatient setting limit immediate generalizability. We also excluded patients with clinical deterioration or adverse trajectories which may limit generalizability to these subgroups. Post-hoc application of the model to these patients showed the absence of the typical improvement pattern, but the small subgroup size precludes definitive conclusions. Importantly, our model was trained and evaluated based on hospital admission vs. discharge status, without additional objective measures of cardiac congestion such as pulmonary artery pressure or serial NT-proBNP. Consequently, we cannot confirm which specific physiological correlate the model captures, and the classification of hospitalization phase could potentially also reflect other clinical factors that differ between admission and discharge. Further mechanistic validation (e.g. laryngostroboscopy) might clarify the physiological basis of acoustic changes observed in ADHF patients and determine whether they relate to specific aspects of clinical recovery. Our design did not include post-discharge follow-up; as residual congestion at discharge remains common, extending monitoring beyond hospitalization would allow more comprehensive evaluation of the complete recompensation cycle and associated voice biomarker changes. For successful outpatient translation, algorithm validation under typical home-recording conditions is required. Future studies should include longer follow-up with defined clinical outcomes (e.g. HF hospitalizations) to assess algorithm impact on clinical management and patient outcomes. Cross-device evaluation was conducted in a relatively small cohort; confirmation in larger, more diverse populations is essential before clinical implementation.

## Conclusion

In this prospective multicentre pilot study, we developed and evaluated a voice- and breathing-based machine-learning approach that discriminates hospitalization phase (admission vs. discharge status) in patients hospitalized with ADHF and captures progressive changes during the inpatient course. Integrating respiratory acoustic features with vowel-derived voice measures improved classification performance compared with voice-only models. Given its non-invasive nature and low patient burden, this approach warrants further investigation as a complementary monitoring signal. Future studies incorporating objective congestion markers (e.g. serial natriuretic peptides, lung ultrasound, radiographic congestion scoring, or invasive haemodynamics where feasible) are required to establish physiological specificity and clinical utility.

## Supplementary Material

ztag052_Supplementary_Data

## Data Availability

Raw voice recordings cannot be shared due to data privacy constraints and institutional approvals. De-identified clinical data, aggregate results, feature-level data, and analysis code may be made available to qualified academic researchers upon reasonable request, subject to approval by the responsible institutional review boards.
