# Supplementary material for: A pilot study on AI-based voice analysis for monitoring patients hospitalized with acute decompensated heart failure
Source: Eur Heart J Digit Health. 2026 Mar 30;7(6):ztag052. doi: 10.1093/ehjdh/ztag052 (PMC13326637; doi:10.1093/ehjdh/ztag052)
Supplement: ztag052_Supplementary_Data [file ztag052_supplementary_data.docx]

**Supplementary material**

**A.0 Exemplary illustration of the recording setup**


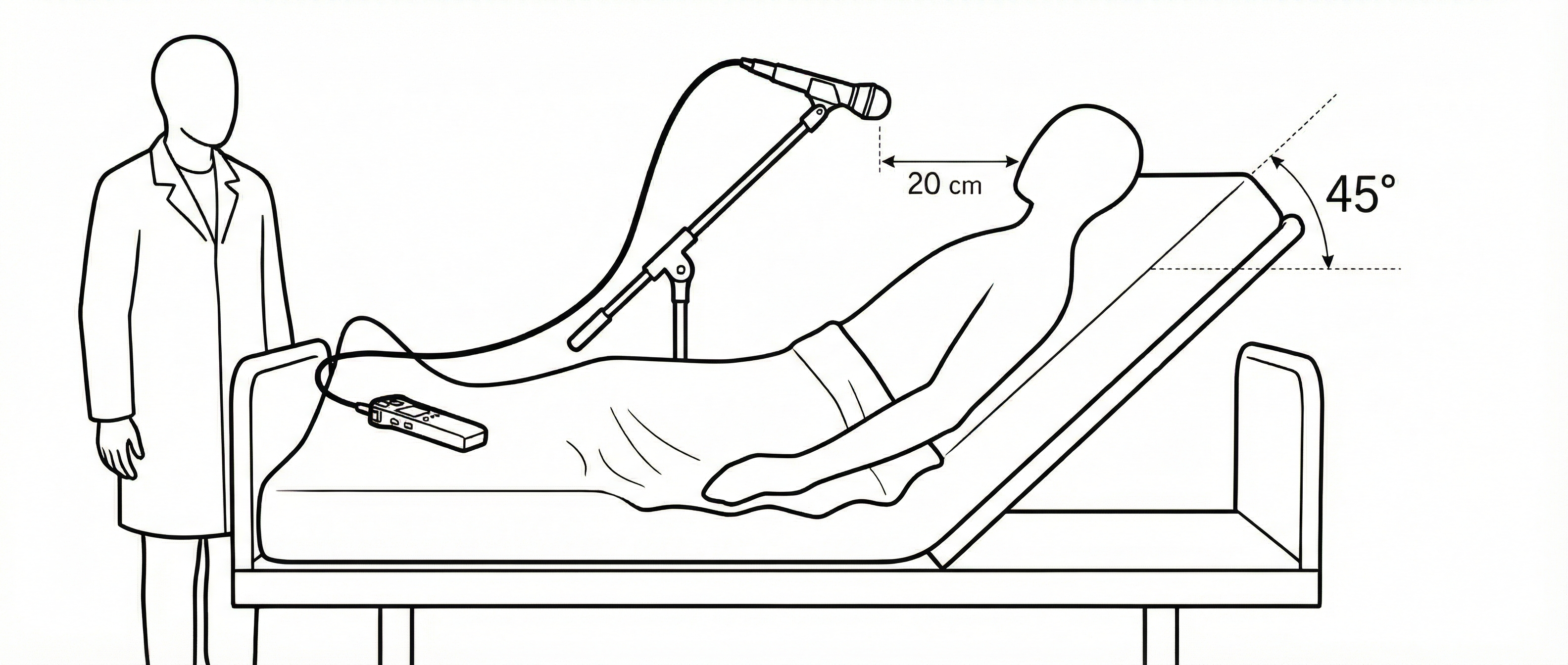
**Supplementary Figure 1.** Voice recording setup. Schematic illustration of the standardized recording setup. Patients were positioned at a 45-degree incline with a directional microphone (Sennheiser MKE 600) placed approximately 20 cm from the mouth. Recording sessions were supervised by clinical investigators.

**A.1 Automated Breathing Segmentation Pipeline**

Initially, a pre-emphasis filter was applied to enhance higher-frequency components, followed by the computation of short-time RMS Energy using 4096-sample frames with a 512-sample hop size. A dynamic, recording-specific threshold was then calculated to detect low-energy regions potentially corresponding to breathing activity. To improve temporal accuracy, detected breathing segments were refined by trimming the first 150 ms and the last 50 ms of each segment. Additionally, regions exhibiting high onset strength - indicating sudden or prominent acoustic change and defined as exceeding the 85th percentile of the onset envelope - were excluded to ensure that only acoustically stable breathing intervals were retained. Finally, segments shorter than 300 ms were removed to eliminate spurious detections. All automatically detected segments were subsequently subjected to manual inspection to confirm the presence of respiratory activity and to exclude artifacts or ambiguous events. An exemplary image for the automated breathing segmentation is demonstrated in Supplementary Figure 2.


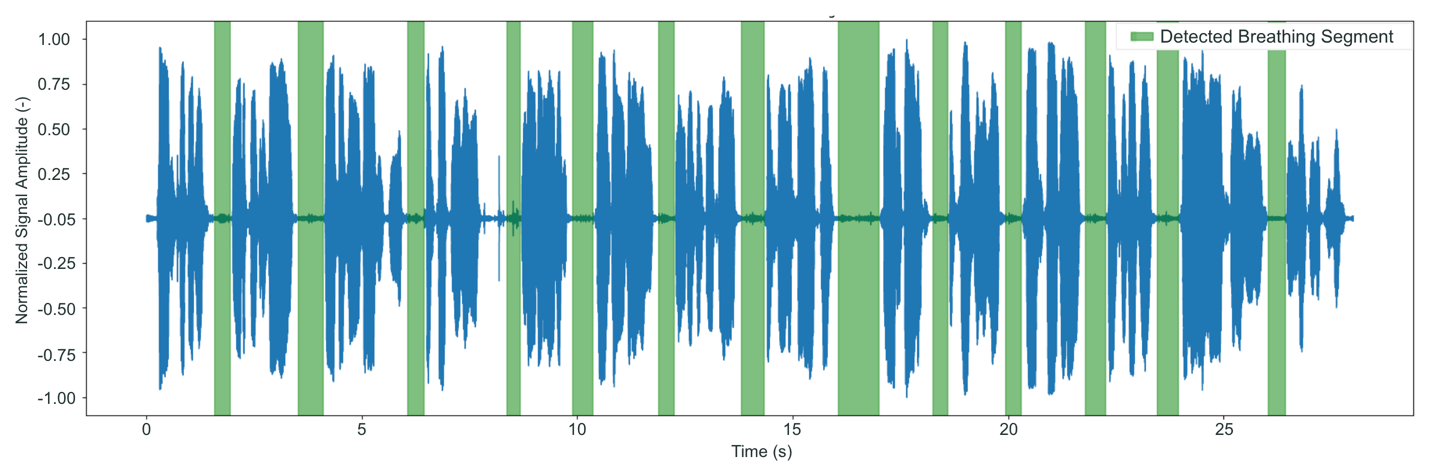
**Supplementary Figure 2.** **Automated detection of breathing segments overlaid on the normalized audio waveform.** A pre-emphasis filter was applied to boost high-frequency content, and short-time RMS Energy was computed using 4096-sample frames with a 512-sample hop size. Low-energy regions were detected using a dynamic threshold and refined by trimming 150 ms from the start and 50 ms from the end of each segment. Regions with onset strength above the 85th percentile were excluded, and segments shorter than 300 ms were discarded. The remaining segments were manually reviewed. Red shaded areas denote validated breathing intervals.

**A.2 Volume-level sensitivity analysis**

In this evaluation, we examined the influence of recording volume on the model performance as a technical sensitivity analysis. A key challenge in this setting is that recording volumes vary across patients, not because of clinical factors but due to technical aspects of the acquisition setup (e.g., external microphone placement and occasional signal dropouts). To quantify how this variability in acquisition-related factors influences performance, we conducted a stepwise analysis in which patients were progressively added based on the root-mean-square (RMS) energy of their recordings.

At the most restrictive threshold (> –35 dBFS (36)), the cohort consisted of 54 patients and served as the baseline. At each subsequent threshold (–50, –55, –60, –65, and –72 dBFS), additional patients were introduced, increasing the cohort size to 66, 70, 77, 79, and 80 patients, respectively. For each expanded dataset, the algorithm was recomputed and the corresponding F1 score was obtained. Model performance decreased with the inclusion of lower-volume recordings, from 0.89 at baseline to 0.79 at the lowest threshold. The proportion of newly introduced low-volume recordings at each step was 0%, 18.8%, 23.5%, 30.6%, 32.5%, and 33.3%, respectively.

This analysis is conceptually separate from the interpretation of loudness variability and spectral flux, which capture relative temporal and spectral dynamics within each normalized recording and are therefore independent of absolute signal amplitude.


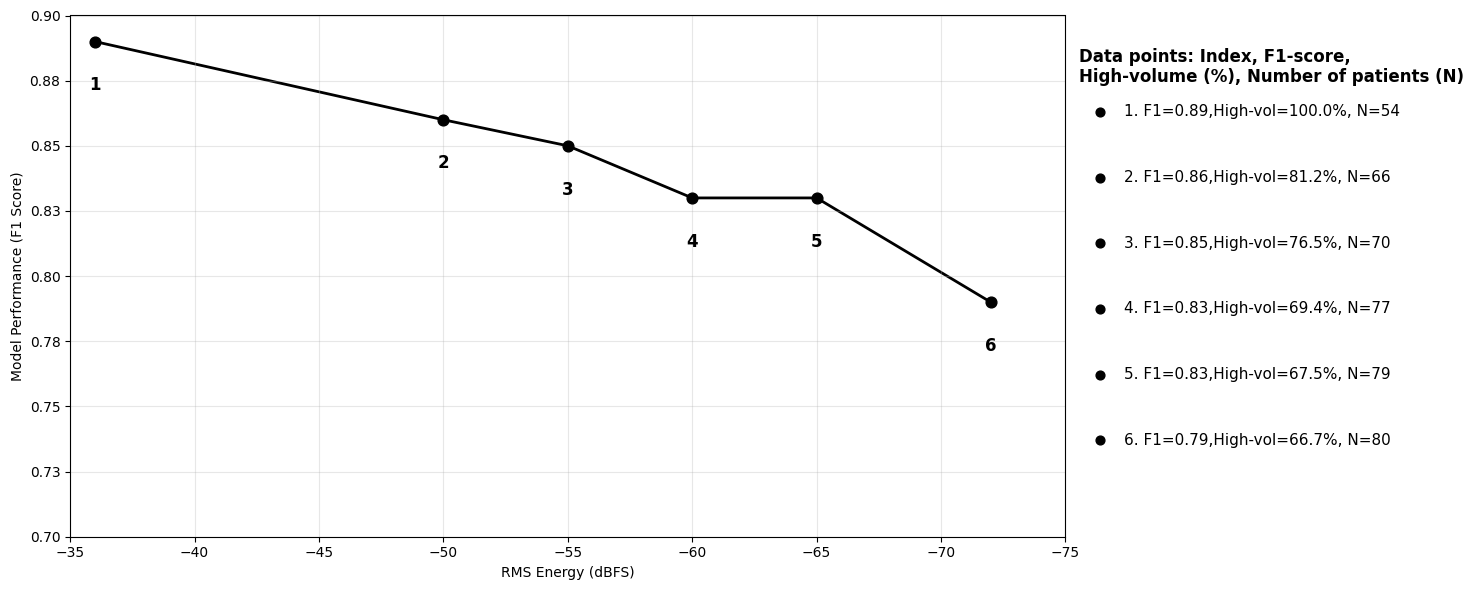
 **Supplementary Figure 3**. Model performance across RMS energy thresholds. The F1 score decreased as progressively lower-volume recordings were introduced.

**A.3 Inspiratory Rise Time Calculation**

To quantify the airflow dynamics during inspiration in breathing sounds, we analysed the envelope of each audio segment to identify the inspiratory rise phase; the portion of the signal during which the amplitude increases from the start of inspiration to its peak.

First, each audio segment was pre-processed by removing its DC offset and applying full-wave rectification to obtain a magnitude-only representation of the signal. The resulting signal was then smoothed using a moving average filter to compute the signal envelope, which reflects amplitude fluctuations associated with airflow during breathing. This envelope representation is more robust to high-frequency noise and phase variations than the raw waveform, making it suitable for analyzing the inspiratory airflow dynamics. To isolate the rising portion of the inspiration, we first identified two key time points in the envelope:

- **The start of inspiration,** defined as the first local minimum of the envelope.
- **The peak of inspiration**, defined as the global maximum of the envelope.

The segment between these two points represents the full inspiratory rise phase. Within this segment, we further isolated the sub-segment where the envelope amplitude increased from 10% to 90% of its peak. This interval was then used to calculate the inspiratory rise slope as an indicator of inspiratory effort, by fitting a linear model to the envelope values within this range. This approach minimizes the influence of noise and plateauing behaviour near the peak, capturing the most dynamically relevant portion of the inspiration. To visualize this process, Supplementary Figure 4 illustrates the steps on a representative breathing segment. The raw signal, its envelope, the 10–90% interval, and the linear fit are shown. This same procedure was repeated for all segments across participants, and the resulting slopes were used in subsequent cohort-level comparisons between admission and discharge conditions.


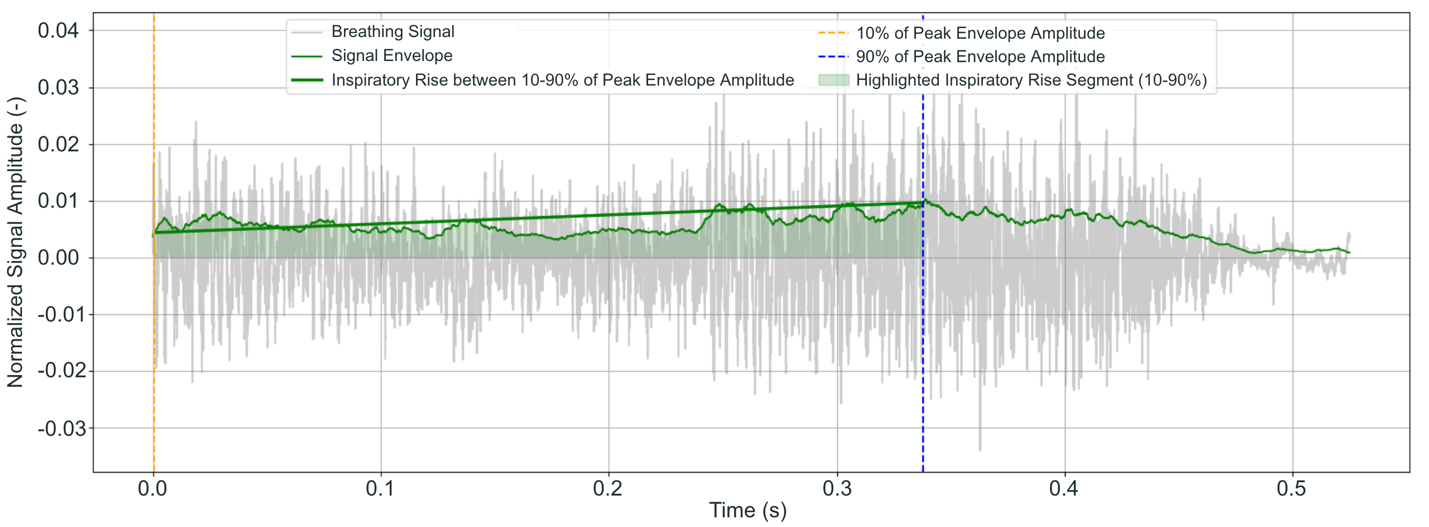
**Supplementary Figure 4***.* **Illustration of the inspiratory rise phase within a representative breathing segment.**

The grey trace denotes the normalized raw audio signal, while the dark green curve represents the smoothed signal envelope. The vertical dashed lines indicate the time points at which the envelope reaches 10% (orange, time = 0.0007 s) and 90% (blue, time = 0.3378 s) of the inspiratory peak amplitude. The shaded green region highlights the segment of the envelope corresponding to this 10–90% amplitude interval. A linear fit across this segment (dark green line) is used to quantify the slope of the inspiratory rise, serving as an indicator of inspiratory effort.

**A.4 Inter-device Audio Signal Variability**

I) Feature analysis: To evaluate the comparability of audio signals recorded by the tablet and professional recorder devices, we performed a statistical analysis on the extracted acoustic features using matched recordings of the same patient performing simultaneously the same audio task on both devices. This ensures a controlled comparison where the only difference is the recording device. We analysed the same feature sets from OpenSMILE as used previously, applying the Wilcoxon signed-rank test along with false discovery rate correction to account for multiple comparisons. Recordings were first aggregated by patient and task by computing the mean of each feature before statistical testing.

Acoustic features had similar distributions across devices, with no substantial differences in mean values or effect sizes. Notably, the features previously found to be significant for distinguishing between admission and discharge, such as spectral flux, jitter, shimmer, did not show statistically significant differences between the two recording devices. This suggests that these features are relatively robust to variations in the recording setup, and consequently, models trained on data from one device type (e.g., professional recorder) are expected to generalize well to recordings from tablets.

Below is a summary table of acoustic features, ordered by their p-values in descending order.

**Supplementary Table I. Statistical Significance of Acoustic Features Across Recording Devices (Tablet vs. Professional Recorder).**

| **Acoustic Feature** | **P-value (Tablet vs. Professional Recorder)** |
| --- | --- |
| Fundamental Frequency | 0.78 |
| Spectral Flux | 0.72 |
| F2 (Formant 2) | 0.72 |
| Zero Crossing Rate | 0.75 |
| Envelope of Fundamental Frequency | 0.60 |
| MFCCs (1–5) | 0.53 |
| F1 (Formant 1) | 0.51 |
| F3 (Formant 3) | 0.27 |
| Shimmer | 0.16 |
| Jitter | 0.088 |

II) Cross-device direct testing:

Professional recordings were acquired at a 96 kHz sampling rate and analysed using 100 ms windows. Because tablet-based recordings were collected at a lower sampling rate (44 kHz), the professional data were downsampled to 44 kHz and re-segmented into 250 ms windows to reduce, though not eliminate, the acoustic differences between recording platforms. This adjustment was applied only for the cross-device validation analysis.

For model training and testing, we employed the same method described in the manuscript, a leave-one-patient-out (LOPO) testing strategy on the main dataset, excluding patients with dual recording setups. Each LOPO model was then tested on the dual-setup cohort, which provided recordings from both professional and tablet devices. Model performance was reported as the average F1 score of all the LOPO models across patients in this dual-device testing scenario.

**A.5 Post-hoc model performance on excluded patients**

To explore whether the admission-to-discharge acoustic pattern observed in the primary cohort was present in patients excluded from the primary analysis, the trained model was applied without retraining to excluded patients with adverse or unclear clinical trajectories. Of the 5 patients excluded for clinical deterioration (ICU/IMC transfer, urgent surgery, or in-hospital death), 4 had sufficient recordings for model evaluation; 1 lacked usable recordings as the hospitalization stay was ≤2 days. All 3 patients excluded for net weight gain at discharge had sufficient recordings and were used for the subsequent analysis.

**Supplementary Table II. Post-hoc model performance on excluded patients.**

| **Subgroup** | **N** | **F1-score** | **95% CI** |
| --- | --- | --- | --- |
| Clinical deterioration (ICU/IMC, urgent surgery transfer, or death) | 4 | 0.40 | 0.06 - 0.73 |
| Patients with net weight gain at discharge | 3 | 0.00 | 0.00 - 0.00 |

**A.6 STROBE Checklist and Study Flow**

Study design: Prospective multicentre observational cohort study.

| STROBE Item | Recommendation | Location in Manuscript (Section) |
| --- | --- | --- |
| 1(a) | Indicate study design in title/abstract. | Title and Abstract |
| 1(b) | Provide informative and balanced abstract. | Abstract |
| 2 | Explain scientific background and rationale. | Introduction |
| 3 | State objectives and prespecified hypotheses. | End of Introduction |
| 4 | Present key elements of study design early. | Methods – Study Design |
| 5 | Describe setting, locations, relevant dates. | Methods – Study Design and Patient Population |
| 6(a) | Eligibility criteria and participant selection. | Methods – Participants |
| 7 | Define outcomes, exposures, predictors, confounders. | Methods – Data Collection, Feature Extraction, and Statistical Analysis |
| 8 | Data sources and measurement methods. | Methods – Voice Recording Protocol & Clinical Data |
| 9 | Describe efforts to address bias. | Methods – Statistical Analysis & Limitations |
| 10 | Explain how study size was determined. | Methods – Study Design and Patient Population |
| 11 | Explain handling of quantitative variables. | Methods – Statistical Analysis |
| 12(a) | Describe all statistical methods. | Methods – Statistical Analysis |
| 12(b) | Methods for subgroups/interactions. | Methods – Dataset Construction and Sensitivity Analyses |
| 12(c) | Explain handling of missing data. | Methods – Dataset Construction; Results – Participant Flow |
| 13(a) | Report participant numbers at each stage. | Results – Participant Flow |
| 13(b) | Reasons for non-participation. | Results – Participant Flow |
| 13(c) | Trial Flow Diagram. | Supplement |
| 14(a) | Participant characteristics. | Results – Baseline Characteristics |
| 14(b) | Missing data per variable. | Results – Baseline Characteristics / Supplement |
| 15 | Outcome events or summary measures. | Results – Model Performance |
| 16(a) | Unadjusted estimates and precision. | Results – Performance Metrics with 95% CI |
| 17 | Other analyses (subgroups/sensitivity). | Results – Subgroup & Cross-Site Analyses |
| 18 | Summarise key results. | Discussion – First Paragraph |
| 19 | Discuss limitations and potential bias. | Discussion – Limitations Section |
| 20 | Cautious overall interpretation. | Discussion – Interpretation Paragraph |
| 21 | Discuss generalisability. | Discussion – Generalisability / Cross-Site Validation |
| 22 | Funding source and role of funders. | Funding Statement |

**
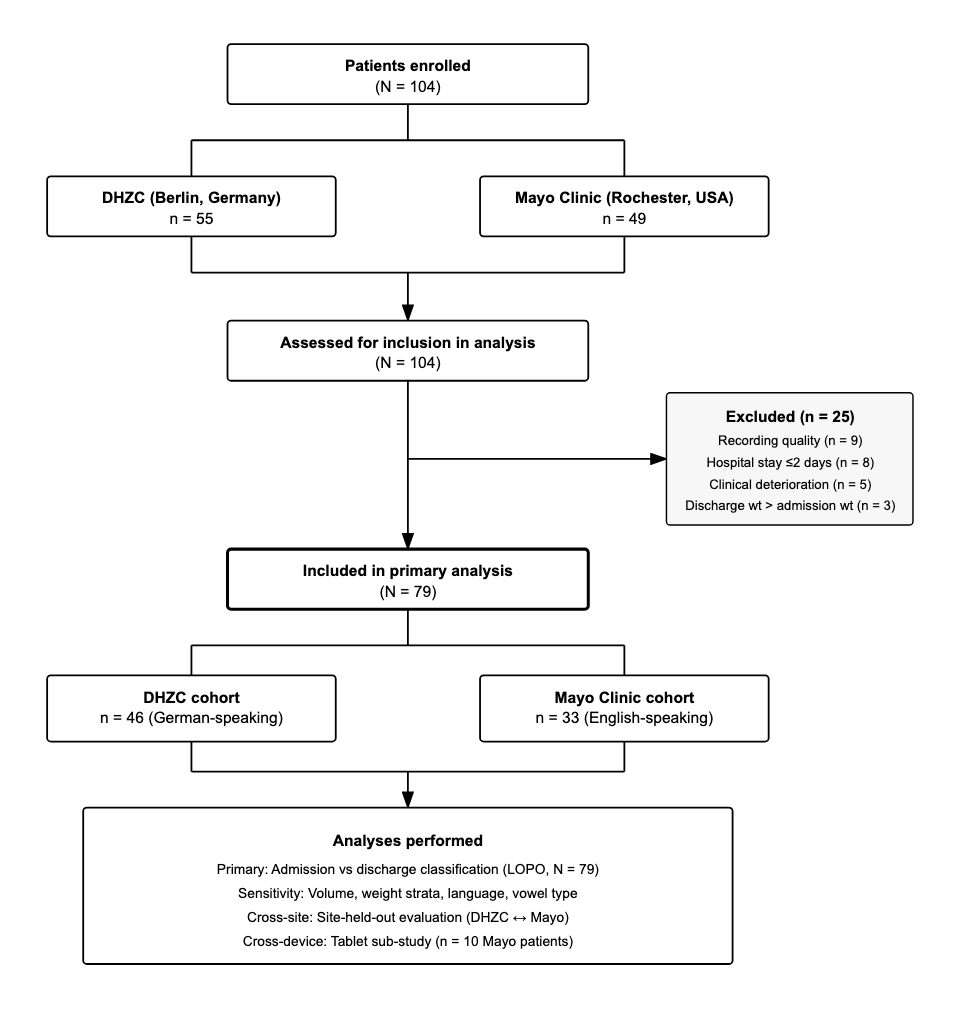
**

**Supplementary Figure 5***.* **Flow chart of the proposed approach for Tracking Decompensation with AI-Based Voice Monitoring in Patients Hospitalized with Acute Decompensated Heart Failure**
